# Thyroidectomy in a Surgical Volunteerism Mission: Analysis of 464 Consecutive Cases

**DOI:** 10.1155/2019/1026757

**Published:** 2019-11-28

**Authors:** Rifat Latifi, Mahir Gachabayov, Shekhar Gogna, Renato Rivera

**Affiliations:** ^1^Department of Surgery, Westchester Medical Center, Valhalla, NY 10595, USA; ^2^Department of Surgery, New York Medical College, Valhalla, NY 10595, USA; ^3^Operation Giving Back, Bohol, Philippines; ^4^Department of Surgery, St. Joseph Hospital, Breese, IL 62230, USA

## Abstract

Although surgical volunteer missions (SVMs) have become a popular approach for reducing the burden of surgical disease worldwide, the outcomes of specific procedures in the context of a mission are underreported. The aim of this study was to evaluate outcomes and efficiency of thyroid surgery within a surgical mission. This was a retrospective analysis of medical records of all patients who underwent thyroid surgery within a SVM from 2006 to 2019. Postoperative complication rate was the safety endpoint, whereas length of hospital stay (LOS) was the efficiency endpoint. Serious complications were defined as Clavien–Dindo class 3–5 complications. Expected safety and efficiency outcomes were calculated using the American College of Surgeons National Surgical Quality Improvement Program (NSQIP) surgical risk calculator and compared to their observed counterparts. A total of 464 thyroidectomies were performed during the study period. Mean age of the patients was 40.3 ± 10.8 years, and male-to-female ratio was 72 : 392. Expected overall (*p*=0.127) and serious complication rates (*p*=0.738) were not significantly different from their observed counterparts. Expected LOS was found to be significantly shorter as compared to its observed counterpart (0.6 ± 0.2 vs. 2.5 ± 1.0 days; *p* < 0.001). This study found thyroid surgery performed within a surgical mission to be safe. NSQIP surgical risk calculator underestimates the LOS following thyroidectomy in surgical missions.

## 1. Introduction

Almost two-thirds of the world's population do not have access to safe, affordable, and timely surgical care, with the majority being from middle-income and low-income countries. It is well recognized that surgical volunteerism missions (SVMs) offer a flexible and often (as in cases of disasters and other situations) rapid response to surgical care worldwide [1]. Many surgeons and different medical institutions and non-for-profit organizations from around the developed world participate in SVMs. Yet, the number of those who participate in SVM is not known and perhaps insufficient. The reason for not getting involved in SVM is many, but one of them is institutional support of the academic surgeon to provide such outreach. A recent position paper based on a cross-sectional survey conducted by a task force from the Association for Academic Surgery Global Affairs Committee and the Society for University Surgeons Committee on Global Academic Surgery reported that academic global surgery involvement represents a very small portion of underrecognized scholarship activity [2]. Operation Giving Back (OGB) program of the American College of Surgeons (ACS) provides a comprehensive platform for volunteers, partners, philanthropists, policy makers, and the public to identify opportunities for surgical volunteer mission [3].

Although safety and efficiency of SVMs carrying out various surgical subspecialties have been reported [4–6], thyroid surgery performed in the context of a surgical mission is underreported [7, 8]. This study represents a large cohort of patients undergoing thyroid surgery from a continuous single multispecialty SVM operated over 13 surgical missions (2006–2019) in one of the islands of Bohol in the Philippines (with a population of 1.3 million people). The Philippines is known to still have iodine deficiency leading to endemic goiters, thyroid cancers, and mental disorders (in newborns) [9], despite attempts to improve such issues through government policies [10]. In fact, the iodine deficiency rate decreased from 65.4% of the population in 1998 to 23.8% in 2003 as a result of such government policies [10].

SVM has been organized over the years by the same group of surgeons and the local support system in the same hospital for 12 out of 13 missions (one mission was organized in a governmental hospital). Recently, we reported a cumulative result of 12 missions on 1,327 operations that were performed (842 females (63.4%) and 485 males (36.6%)) of which the majority of operations were for thyroid disease (31.6%), followed by hernia (17.3%), hysterectomies/salpingo-oophorectomies (12.2%), soft tissue tumors (9.9%), cleft lip/palate repairs (7.2%), breast (6.4%), gallbladder disease (4.7%), cataract (2.9%), parotid masses (1.4%), and others (6.4%) [11].

The aim of this study was to evaluate the safety and efficiency of thyroid surgery performed within a surgical mission, as one of the most common procedures performed in this mission.

## 2. Materials and Methods

### 2.1. Study Design, Eligibility Criteria, and Collected Data

A retrospective analysis of prospectively collected data of the patients who underwent thyroid surgery during the thirteen missions held between 2006 and 2019 was performed. Inclusion criterion was adults older than 18 who underwent thyroid surgery during those missions. The data were collected to predefined Microsoft Excel spreadsheets and were stored within the headquarters of the mission, Borja Hospital, Tagbilaran, Philippines. Collected data comprised patients' demographics and intra- and postoperative variables. The study was approved by the Institutional Review Board of the University of Arizona, as a part of a larger study [11]. The study report complies with the STROCSS criteria [12].

### 2.2. Study Endpoints

Safety endpoints observed were overall postoperative complication rate and postoperative serious complication rate as compared to their expected counterparts. Efficiency endpoint observed was length of hospital stay as compared to its expected counterpart.

### 2.3. Mission

“Operation Giving Back Bohol” (OGBB) is a week-long SVM which was organized in the province of Bohol, located in the Central Visayas region of the Philippines in 2006, and held annually (except for 2008). Volunteers from different US institutions including surgeons of various disciplines (general surgery, plastic surgery, otorhinolaryngology, and gynecology), anesthesiologists, nurse anesthetists, surgical and anesthesia residents, surgical nurses, surgical technologists, and others took part in these missions. The OGBB mission was supported by a number of organizations, the local government, and individuals from the community and a nongovernmental organization Gift of Life™ Foundation for the Philippines. Thyroidectomies were performed by general surgeons, surgical oncologists, and ENT surgeons (on occassion).

### 2.4. Patient Selection and Preoperative Evaluation

Gift of Life™ Foundation in cooperation with the physicians of a local hospital screened, identified, and evaluated patients requiring surgery including thyroid surgery. Preoperative workup including basic biochemical analysis, thyroid function test, chest XR, cytology of thyroid masses, and medical clearance were performed in the local hospital. On occasion, a CT scan of the neck and chest was performed for potentially difficult airways and to assess relationship of the mass with the vascular structures and esophagus. On the day of surgical team's arrival, as many as 200 patients in a single day are screened. After evaluating the patients, surgeries were scheduled for six operative days (Sunday–Friday). Store-and-forward telemedicine was utilized as appropriate to improve cooperation in recent years in order to better prepare for the mission as well as communicate with local surgeons in case of complications occurring after the team had left [13].

### 2.5. Study Interventions

All procedures were performed under general anesthesia after obtaining patients' consent. Thyroid surgery included partial, subtotal, and total thyroidectomies depending on the type and extent of the disease. In case of thyroid malignancies involving regional lymph nodes, cervical lymphadenectomy was performed in addition to thyroidectomy. After surgery, the patients were treated in a PACU and discharged to the general ward as appropriate. Discharge home criteria included no evidence of complications, ambulation and ability for self-care or good family support, tolerance of oral intake, removal of drains (when present), and adequate pain control with oral analgesia. Postoperative patients are seen daily by the operating team. Calcium levels were not checked in all patients since all patients undergoing subtotal or total thyroidectomy were supplemented with calcium gluconate tablets, while in the hospital, and advised to continue with calcium therapy.

### 2.6. Definitions

Body mass index was expressed in kg/m^2^. Physical status of the patients was classified according to the American Society of Anesthesiologists (ASA) score. Postoperative complications were defined as any deviation from the normal postoperative course and classified according to the Clavien–Dindo system [14]. Postoperative serious complication was defined as any complication categorized as a Clavien–Dindo class higher than 2.

### 2.7. Statistical Analysis

American College of Surgeons National Surgical Quality Improvement Program Surgical Risk Calculator (ACS-NSQIP SRC) was used to evaluate the predicted risk of complications, serious complications, mortality, reoperation, and predicted length of hospital stay [15]. Statistical analysis was performed using SPSS software (version 18; SPSS Inc., Chicago, IL, United States). To calculate an expected rate, the mean risk of an outcome was calculated. The data were tested for normality using Kolmogorov–Smirnov and Shapiro–Wilk tests and histograms. Mean with standard deviation and median with interquartile range were used as descriptive statistics for continuous variables. Categorical variables were expressed in numbers, percentages, or ratios. Student's *t*-test was used to compare continuous variables, and chi-squared test was used to compare categorical variables. Statistical significance was defined as *p* < 0.05.

## 3. Results

Four hundred and sixty-four patients underwent surgery for thyroid disease during the thirteen missions between 2006 and 2019. Male-to-female ratio was 72 : 392. Baseline characteristics are shown in Table 1. The indication for surgery in most patients was benign disease, predominantly goiter; however, in the last years of the mission, there was an increase of malignancies or suspected malignancy in cytology reports taken by a local pathologist. Intra- and postoperative complications are shown in Table 2. Fifty-four (12%) total and 56 (12%) subtotal thyroidectomies were performed. The rest (76%) were partial thyroidectomies. There were five intraoperative complications (blunt injury of the recurrent laryngeal nerve in 2 patients requiring temporary tracheostomies, bleeding in 2 patients, and bradycardia in one patient). The overall postoperative complication rate was (13/464) 2.8%. One patient required exploration of the neck for a hematoma. There was one death, after the family withdrew care in a patient that underwent toilet thyroidectomy.

**Figure 1:**
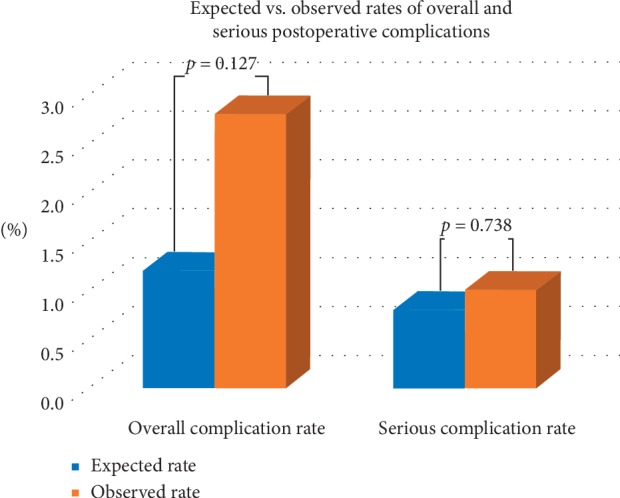
Expected vs. observed postoperative overall and serious complication rates.

Figure 1 depicts the safety endpoint of the study. The expected complication rate was found to be 1.2% (6/464) using the ACS-NSQIP SRC. Expected vs. observed complication rate was not found to be significantly different (*p*=0.127). Expected serious complication rate was found to be 0.9% (4/464) which was not significantly different from its observed counterpart (*p*=0.738).

**Figure 2:**
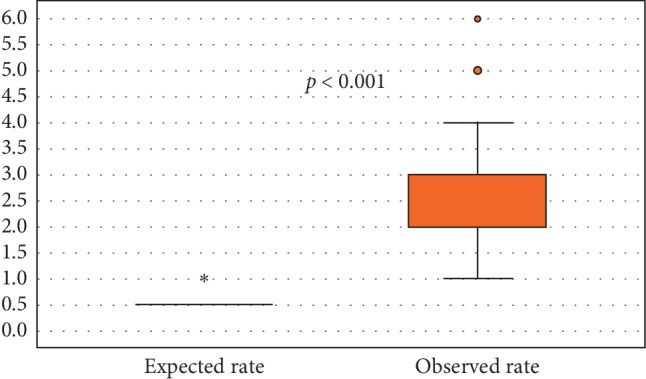
Expected vs. observed length of hospital stay (days).

Figure 2 shows the efficiency endpoint of the study. Expected LOS was found to be significantly shorter as compared to its observed counterpart (0.6 ± 0.2 vs. 2.5 ± 1.0 days; *p* < 0.001).

## 4. Discussion

The main finding of this retrospective cohort study was that thyroid surgery within a surgical mission is safe with comparable overall and serious postoperative complication rates with those predicted by the ACS-NSQIP SRC. Efficiency, however, was found to be lower than expected under normal conditions. Since its development, the abovementioned tool for surgical risk assessment is widely used for decision support or quality control in different settings [16]. A recent retrospective cohort study involving 298 and 138 patients undergoing thyroid and parathyroid surgery, respectively, reported discrepancy between ACS-NSQIP SRC predicted and observed rates of reoperation, emergency room visits, and readmissions [17]. To the best of our knowledge, that was the only study where ACS-NSQIP SRC was utilized in thyroid surgery. Another recent study reporting the outcomes of US Navy's Pacific Partnership mission used ACS-NSQIP SRC in the setting of a surgical mission and found that the calculator overpredicted postoperative morbidity risk (2.0% vs. 0.7%) [18]. However, the authors concluded that the calculator offers a good starting point for humanitarian surgery risk calculation.

Postoperative complication rates in our cohort corroborate with those reported previously in two studies based on the NSQIP database [19, 20]. In fact, the postoperative complication rate in inpatients after thyroid surgery was reported to be 5% as compared to 3% in outpatients [21]. Independent predictors of morbidity in patients undergoing thyroid surgery include age >70, non-Caucasian race, dependent functional status, and comorbidities, such as diabetes, chronic obstructive pulmonary disease, hypertension, dialysis, congestive heart failure, bleeding disorders, and preoperative sepsis. In addition to them, malignant thyroid disease and surgical approach (total thyroidectomy) were found to be independent predictors of postoperative morbidity [19]. Octogenarians were found to have 2.7 times higher risk of postoperative morbidity [21]. Length of hospital stay is controversial in the medical literature. Same day surgery was challenged in several previous publications from different countries [22–24]. A recent meta-analysis including 14 observational studies totaling 10,526 patients reported that discharging patients the same day following a thyroidectomy procedure is as safe and effective as admitting them for observation [25]. Decreasing the length of stay or implementing the same day surgery in the setting of a surgical mission was impossible in our case due to the lack of outpatient care component in our mission team.

Such surgical missions were reported to be “very cost effective” per WHO parameters in a study evaluating cost efficiency of surgical missions in Ghana [26]. The authors found the cost per one averted disability-adjusted life year (DALY) to be 136.5 USD. In fact, 5% of patients only in that cohort underwent thyroid surgery. Another group reported the cost per averted DALY to be 304 USD in a surgical mission held in the Dominican Republic [27]. Evaluating the cost benefit of our mission will be one of the next steps in the evaluation of this surgical mission.

**Figure 3:**
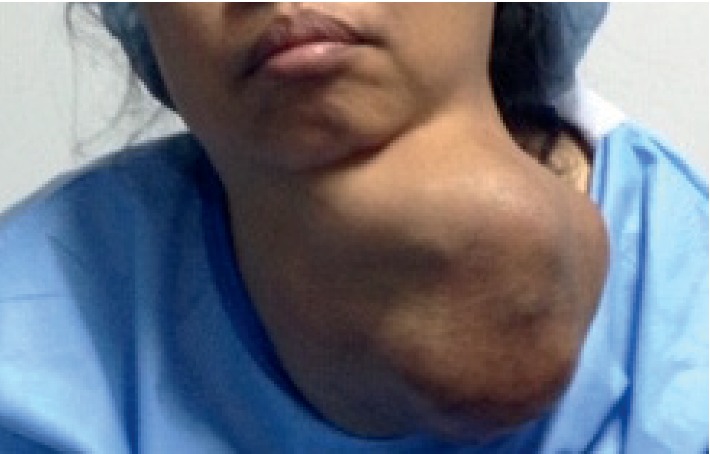
Recurrence of papillary carcinoma of the thyroid gland a year after thyroidectomy.

**Figure 4:**
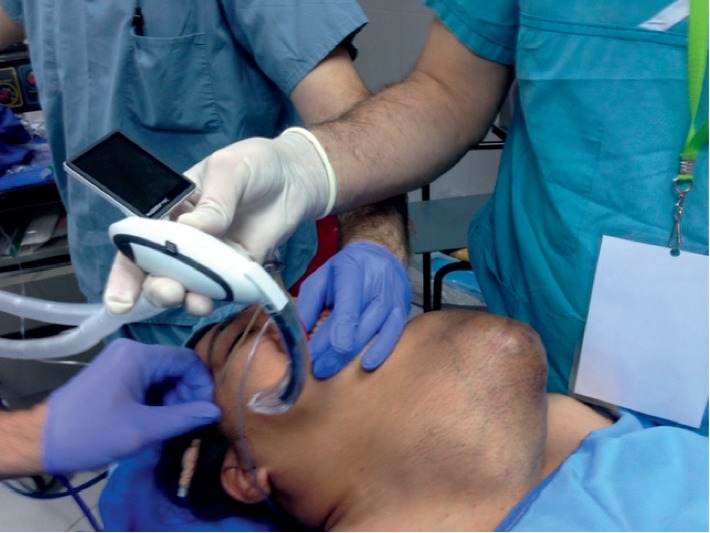
Neglected goiter presenting with difficult airways.

**Figure 5:**
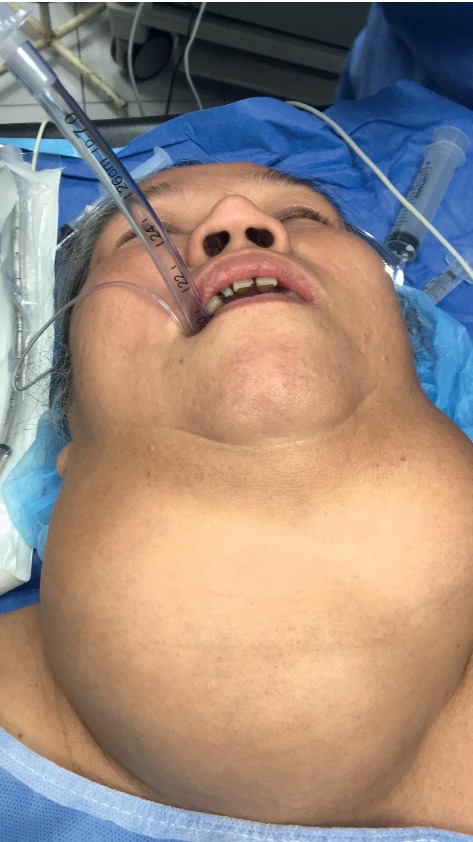
Neglected goiter, a preoperative view.

**Figure 6:**
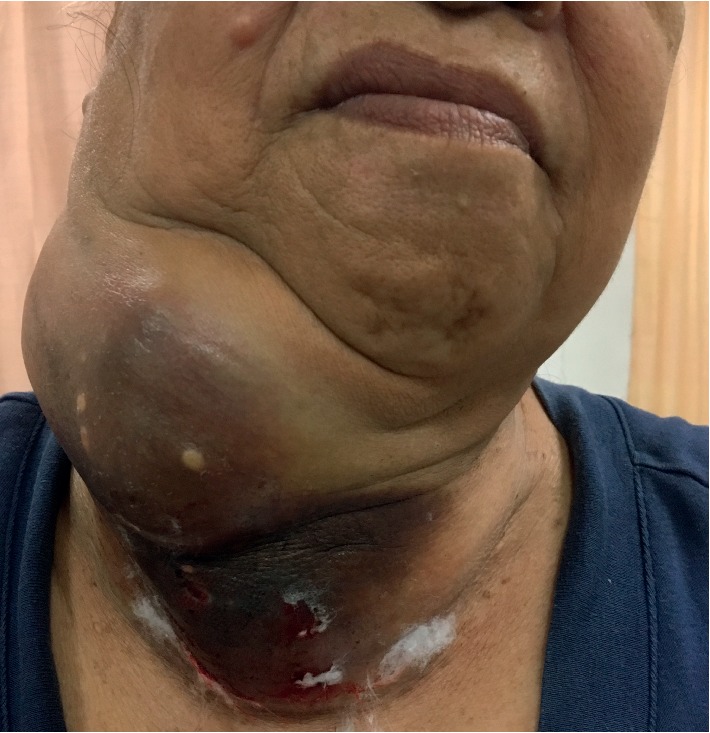
Neglected thyroid cancer.

In a recent paper, we have outlined the major challenges of surgical mission, which we have divided broadly into seven groups: (1) creation of the team; (2) patient selection; (3) case complexity; (4) cost of the mission; (5) quality of care and safety of both patients and the team; (6) changing the mindset and situational awareness; and (7) overall efficiency of the SVM [11]. However, when it comes to thyroid surgery and in particularly thyroid postoperative care, in addition to being complex even in the best of conditions there are other challenges that we have to take into consideration. Each patient undergoing thyroid surgery should have long-term follow-up by the primary care physician and the endocrinologist, as well as the surgeon. Potential complications such as recurrence of tumor (in malignancy cases) (Figure 3) due to lack of follow-up and treatment make thyroid surgery in surgical missions basically rudimentary and “toilet” surgery. While removing the huge masses from the neck, be it for neglected benign disease (Figures 4 and 5) or malignancy (Figure 6), is a major contribution to the patients' well being, and inability to even have a final pathologic report in patients who are suspected to have malignancy or those with documented malignancy (most patients cannot afford it, unless provided by the mission) is seriously worrisome. Moreover, none of these patients is staged appropriately for recurrence, distant metastasis, lymph node metastasis, extrathyroid extension, tumor size, and completeness of resection. In addition, long-term biochemical and metabolic complications such as hypoparathyroidism (in cases of total thyroidectomy) are unknown. Inability of surgeons in SVM to treat and follow-up patients who may require, for example, thyroid ablation for distance metastasis (in case of recurrence), or reoperation is a major drawback of SVMs. So, perhaps this brings a real ethical dilemma of thyroid surgery in surgical missions. While the perioperative safety is team and surgeon based, the inability to follow-up patients long-term is a serious issue.

To this end, we would like to reiterate the limitations of our study. While the data reported here were collected prospectively, the lack of long-term outcome data is the biggest limitation although there were few patients that were seen in follow-up. One of them was a 31-year-old female undergoing “toilet” thyroidectomy who underwent total thyroidectomy and neck dissection but had tumor wrapping the vascular structures in the left side of the neck (Figure 3). Unfortunately, a year later, she had massive recurrence that was inoperable in such a mission. She had no ability to get chemoradiation therapy or any kind of postoperative follow-up. One other patient came back for a scar revision. We have no data on hypocalcemia occurrence, as we supplemented all patients undergoing subtotal or total thyroidectomy with calcium.

## 5. Conclusions

This retrospective cohort study found thyroid surgery performed within a surgical mission to be safe. Efficiency was found to be lower as compared to western conditions as defined by NSQIP. An administrative database would help to better understand the challenges, outcomes, and strategies for the improvement of surgical missions.

## Figures and Tables

**Table 1:** Demographics and preoperative variables.

	All patients (*n* = 464)
Age (years)^*∗*^	40.3 ± 10.8
Gender (*n* (%))	
Male	72 (15.5%)
Female	392 (84.5%)
BMI (kg/m^2^)^*∗*^	22.6 ± 3.3
ASA (*n* (%))	
I	414 (89%)
II	45 (10%)
III	5 (1%)
Comorbidities (*n* (%))	50 (10.8%)
Diagnosis (*n* (%))	
Benign	398 (85.8%)
Malignant	66 (14.2%)
Length of current disease (years)^*∗*^	7.8 ± 6.1
Number of surgeries per year^#^	39 (18–50)

^*∗*^Expressed in mean ± standard deviation; ^#^expressed in median (range). BMI, body mass index; ASA, American College of Surgeons.

**Table 2:** Intra- and postoperative variables.

	All patients (*n* = 464)
Number of surgeries per year^#^	39 (18–50)
Operating time (min)^*∗*^	61.9 ± 26.3
Intraoperative complications (*n* (%))	5 (1.1%)
Overall postoperative morbidity (*n* (%))	13 (2.8%)
Clavien–Dindo 2	8 (1.7%)
Clavien–Dindo 3	3 (0.6%)
Clavien–Dindo 4	1 (0.2%)
Clavien–Dindo 5	1 (0.2%)
Reoperation (*n* (%))	3 (0.6%)
Overall postoperative mortality (*n* (%))	1 (0.2%)
Length of hospital stay (days)^*∗*^	2.5 ± 1.0

^*∗*^Expressed in mean ± standard deviation; ^#^expressed in median (range).

## Data Availability

The raw data used to support the findings of this study are restricted by the Institutional Review Board of the University of Arizona and Borja Hospital in order to protect patient privacy. Data are available from the corresponding author for researchers who meet the criteria for access to confidential data.
